# Sequence effects and speech processing: cognitive load for speaker-switching within and across accents

**DOI:** 10.3758/s13423-023-02322-1

**Published:** 2023-07-13

**Authors:** Drew J. McLaughlin, Jackson S. Colvett, Julie M. Bugg, Kristin J. Van Engen

**Affiliations:** 1 Department of Psychological and Brain Sciences, Washington University in St. Louis, St Louis, MO USA; 2 Basque Center on Cognition, Brain and Language, Paseo Mikeletegi, 69, 20009 Donostia-San Sebastián, Gipuzkoa Spain

**Keywords:** Pupillometry,·Speech processing, Congruency sequence effect, Accent

## Abstract

Prior work in speech processing indicates that listening tasks with multiple speakers (as opposed to a single speaker) result in slower and less accurate processing. Notably, the trial-to-trial cognitive demands of switching between speakers or switching between accents have yet to be examined. We used pupillometry, a physiological index of cognitive load, to examine the demands of processing first (L1) and second (L2) language-accented speech when listening to sentences produced by the same speaker consecutively (no switch), a novel speaker of the same accent (within-accent switch), and a novel speaker with a different accent (across-accent switch). Inspired by research on sequential adjustments in cognitive control, we aimed to identify the cognitive demands of accommodating a novel speaker and accent by examining the trial-to-trial changes in pupil dilation during speech processing. Our results indicate that switching between speakers was more cognitively demanding than listening to the same speaker consecutively. Additionally, switching to a novel speaker with a different accent was more cognitively demanding than switching between speakers of the same accent. However, there was an asymmetry for across-accent switches, such that switching from an L1 to an L2 accent was more demanding than vice versa. Findings from the present study align with work examining multi-talker processing costs, and provide novel evidence that listeners dynamically adjust cognitive processing to accommodate speaker and accent variability. We discuss these novel findings in the context of an active control model and auditory streaming framework of speech processing.

**Supplementary information:**

The online version contains supplementary material available at 10.3758/s13423-023-02322-1.

## Introduction

Listeners often understand spoken language seemingly effortlessly. However, the process of mapping acoustic input onto their linguistic representations can be complicated by individual speaker variability, such that detrimental effects are observed in task blocks with multiple speakers (changing from trial-to-trial) as compared to blocks with a single speaker (Choi et al., 2018; Choi & Perrachione, 2019; Heald & Nusbaum, 2014; Martin et al., 1989; Mullennix et al., 1989). These *multi-talker processing costs* are often attributed to the active control required to achieve phonetic constancy across different talkers (Nusbaum & Magnuson, 1997; Magnuson & Nusbaum, 2007), or disruption of auditory streaming (Kapadia & Perrachione, 2020; Mehraei et al., 2018). In the present study, we examine the costs associated with multi-talker as well as multi-accent processing using pupillometry, and use these costs to inform these two accounts of speech processing.

One account, the *active control model,* proposes that effects of speaker changes reflect the attentional load required to achieve phonetic constancy (Heald et al., 2016). Mechanistically, the load associated with speaker changes reflects engagement of a talker accommodation mechanism that maps each speaker’s idiosyncratic speech to the listener’s phonological space (see schematic representation by Magnuson, 2018). Similarly, exemplar (Johnson, 1997; Pierrehumbert, 2002) and nonanalytic episodes theories (Goldinger, 1998; Nygaard & Pisoni, 1998)^1^ propose that episodes in memory are activated by the incoming acoustic signal during speech processing, improving efficiency. The better performance observed when there is no change in speaker is accounted for by weighting recent episodes more heavily.

A second account is an *auditory streaming framework*, in which speaker changes disrupt selective auditory attention (Shinn-Cunningham, 2008), incurring processing costs as a listener refocuses their attention from one auditory object (a speaker) to another (Kapadia & Perrachione, 2020; Mehrai et al., 2018). Further work suggests that an active control mechanism may support multi-talker speech processing in addition to selective auditory attention (Choi et al., 2022). Listener performance in multi-talker blocks steadily improved when words were preceded by up to 600 ms by the article “a” spoken by the speaker of the upcoming stimulus, consistent with a stimulus-driven reorientation of auditory attention. However, for intervals greater than 600 ms (examined parametrically up to 1,500 ms), no additional benefits were observed, and a baseline processing cost remained for multi-talker blocks at all intervals. These findings suggest that two mechanisms are involved in accommodating speaker changes, and that the mechanisms proposed in the auditory streaming framework and the active control model may operate in parallel.

In the present study, we investigated whether the cognitive demands for switching between speakers of the same accent differed from those for switching between speakers of different accents. A key question is whether a speaker’s familiarity (the familiarity of their accent, specifically) benefits a listener in a multi-talker listening setting. Multiple theories support the hypothesis that more familiar speech ought to be afforded a benefit. Based on an exemplar model, for example, first (L1) language- accented speech ought to be more efficient for an L1 listener to accommodate than unfamiliar second (L2) language-accented speech, because it will be better represented in memory.

The potential benefit of familiarity on multi-talker processing costs has been investigated previously by Magnuson et al. (2021). Based on an active control model (Magnuson & Nusbaum, 2007), Magnuson et al. suggested that multi-talker processing costs may be reduced for familiar speakers because characteristics of their speech would be stored in memory. To test this hypothesis, Magnuson et al. examined the processing costs associated with speaker changes for familiar speakers (family members) versus unfamiliar speakers. The results of the study, however, did not reveal a familiarity benefit for multi-talker processing costs (although benefits were observed in talker identification and speech-in-noise transcription tasks).

We test an extension of Magnuson et al.’s (2021) familiarity benefit hypothesis by examining multi-talker processing costs for familiar and unfamiliar accents. We predicted that systematic and idiosyncratic deviations in how speech is produced by L2 speakers (as compared to L1 speakers) may exacerbate perceptual demands in a multi-talker setting. In other words, accommodating the “phonetic distance” between L1 and L2 speakers’ productions may result in different processing costs than accommodating the “phonetic distance” between two L1 speakers’ productions, making it easier to observe a familiarity benefit. Notably, while processing costs for alternations between speakers can be accounted for by an active control or exemplar model, a familiarity benefit is not accounted for by the auditory streaming framework. Further, the auditory streaming framework would not be able to account for a difference in multi-talker processing costs for switching between speakers of different accents versus speakers of the same accent.

## Pupillometry

Our examination of multi-talker processing costs takes a novel approach – using pupillometry to assess trial-to-trial changes in cognitive processing load. Pupillometry, the measure of pupil diameter over time, has been used across multiple domains as a physiological index of cognitive processing load (Beatty, 1982). By tracking the “task-evoked” pupil response, one can compare the cognitive demands imposed by different tasks or experimental manipulations. In speech processing, cognitive pupillometry has been applied widely (for a review, see Van Engen & McLaughlin, 2018), demonstrating a systematic relationship between the magnitude of the pupil response and intelligibility of noise-degraded (Zekveld et al., 2010; Zekveld & Kramer, 2014) and L2-accented speech (Porretta & Tucker, 2019). For highly intelligible materials (e.g., sentences that are fully understood by the listener), pupillometry has been used to reveal that increasing signal degradation results in larger pupil response (Winn et al., 2015), as does an L2, as compared to an L1, accent (Brown et al., 2020; McLaughlin & Van Engen, 2020).

Multi-talker processing costs have also been examined with pupillometry (Lim et al., 2021). Using concurrent EEG and pupillometry, Lim and colleagues examined the costs associated with performing a delayed-recall digit span task for single versus mixed talker blocks. Interstimulus interval (ISI) was manipulated such that digits were presented either 0 ms or 500 ms apart. The pupillometry data indicated a larger task-evoked response for the mixed-talker blocks compared to the single-talker blocks, but only for the short ISI. The EEG data indicated a P3a neural response associated with multi-talker blocks, a component that has been linked to attentional reorientation (Polich, 2007). Thus, the study demonstrated pupillometry’s sensitivity to multi-talker processing costs for L1-accented speech and indicated that alternating talkers may result in attentional reorientation that is cognitively demanding.

## Sequence effects

While analyzing the effect of trial N-1 on trial N is a somewhat novel approach to assessing the demands of speech processing, it is common in other cognitive science literatures. For example, the congruency sequence effect (CSE; Gratton et al., 1992) refers to a reduction in the performance difference between congruent (e.g., RED in red-colored ink) and incongruent (e.g., RED in blue-colored ink) trials in conflict tasks such as Stroop when the previous trial is incongruent as opposed to congruent (for reviews, see Duthoo et al., 2014a; Egner, 2007). The CSE is interpreted as an adaptive adjustment of control based on the previous trial type, such that upregulating control when trial N-1 is incongruent leads to less susceptibility to conflict on trial N (Botvinick et al., 2001; cf. Schmidt & Weissman, 2014). Sequence effects have been examined for multi-talker processing costs in work by Kapadia and Perrachione (2020). Using a speeded word-identification task, the authors demonstrated that the efficiency of word identification was reduced on trials in which a speaker switch was made, even when the switch was predictable.

## Research questions and hypotheses

We investigated speaker sequence effects using pupillometry to assess three key research questions. First, is there a measurable cost for switching between speakers? Second, does the magnitude of a switching cost depend on the “phonetic distance” between two speakers’ productions? That is, is switching between speakers with the same accent easier than switching between speakers with different accents? Finally, are all across-accent switches equally difficult? That is, will switching from an L1 accent to an L2 accent be equivalent to switching from an L2 accent to an L1 accent, or will the L1 accent be afforded a familiarity benefit?

We report two experiments that serve as an initial test of how the speaker and accent on the previous trial affect cognitive load on the current trial. We predicted that:

1. Cognitive load would be greater when switching speakers than when repeating the same speaker.
2. Switching across accents would be more cognitively demanding than switching within an accent.
3. There would be an interaction, reflecting a familiarity benefit for L1 accent.

## Experiment 1

In Experiment 1, we re-analyzed data from McLaughlin and Van Engen (2020) to examine the effects of switching between an L1 and an L2 speaker of English. Full methodological details can be found in the original paper.

### Method

Pre-registration, materials, experiment, data, and analysis code for McLaughlin and Van Engen (2020) are available from https://osf.io/7dajv/. The current re-analysis of this data was not pre-registered. Data and analysis code are available from https://osf.io/ajmqz.

### Dataset description

The McLaughlin and Van Engen (2020) study recruited a sample of 52 young adult subjects (39 female and 13 male; *M*_*age*_ = 19.46 years, *SD* = 1.07 years) from the Washington University Psychology Participants Pool. All subjects were screened for normal hearing and were L1 speakers of American English with little exposure to Mandarin Chinese.

Subjects’ pupil response was tracked during presentation of sentence-length materials. Two speakers were presented during the session: an L1 American-accented speaker of English, and an L2 Mandarin Chinese-accented speaker of English. Sixty trials were presented (30 per accent) in a randomized order. After each trial, subjects repeated the sentence aloud. Every three trials, using a scale of one to nine, subjects pressed a key to indicate how effortful it was to understand the previous speaker.

Subjects’ responses were scored for recognition accuracy. Any trials in which keywords were missed were excluded from the dataset. Data were pre-processed following standard pupillometry procedures: blinks were identified, expanded, and interpolated across; data were smoothed with a 10-Hz moving average window; data were baselined using the 500 ms of data immediately preceding stimulus onset (i.e., baseline values were subtracted from all values in the respective trial); and data were time-binned, reducing the sampling frequency from 500 Hz to 50 Hz. Trials with more than 50% missing data were excluded from analyses.

### Preparation of dataset for novel analyses

A switch condition was added to the dataset by comparing the current trial’s (N) accent condition against the previous trial’s (N-1) accent condition. If the two trials matched, they were labeled “no switch,” and if they did not match, they were labeled “switch.” Trial 1 data were removed (as there was no preceding context), as were any trials following an excluded trial (i.e., due to blinks or intelligibility). A total of 80 trials (approximately 2.7%) were removed from the original dataset in this process.

### Growth curve analysis

Growth curve analysis (GCA) was implemented with the lme4 R package (Bates et al., 2014) to examine the data. GCA is a mixed-effects modeling approach similar to polynomial regression (Mirman, 2016). Orthogonalized polynomial predictors (linear, quadratic, cubic, etc.) are incorporated into the fixed and random effects of the model, allowing for a non-linear time-course analysis. This approach is frequently used for analyzing pupillometry data because the curve of a task-evoked pupil response can be fit with a polynomial basis (i.e., is often similar to a cubic shape). In GCA, fixed effects of conditions determine whether there are differences in overall magnitude between levels (i.e., shifting the curve vertically), and interactions between these fixed effects and the fixed effects of the polynomial parameters determine whether the shape of the pupil response differs by condition (i.e., does the rate of increase in pupil size differ by condition?). The random effect structure of all models included random intercepts for subjects and items and random slopes of the linear, quadratic, and cubic polynomials nested within subjects and items.

### Results

Table 1 summarizes all log-likelihood model comparisons from the growth curve analysis. The linear, quadratic, and cubic polynomials all significantly improved fit (all *p*s < .001).

**Table 1:** Log-likelihood model comparisons for growth curve analysis of Experiment 1

Effect	*χ*^2^	Df	*p*
Linear polynomial	12609	1	< .001 ***
Quadratic polynomial	4790.60	1	< .001 ***
Cubic polynomial	74.37	1	< .001 ***
Accent (Levels: L1 Accent, L2 Accent)	10.57	1	.001 **
Switch (Levels: No Switch, Switch)	303.43	1	< .001 ***
Accent × Switch	72.94	1	< .001 ***
Accent × Linear polynomial	16.54	1	< .001 ***
Accent × Quadratic polynomial	0.11	1	.74
Accent × Cubic polynomial	1.48	1	.22
Switch × Linear polynomial	0.27	1	.60
Switch × Quadratic polynomial	13.78	1	< .001 ***
Switch × Cubic polynomial	0	1	> .99

**Fig. 1:**
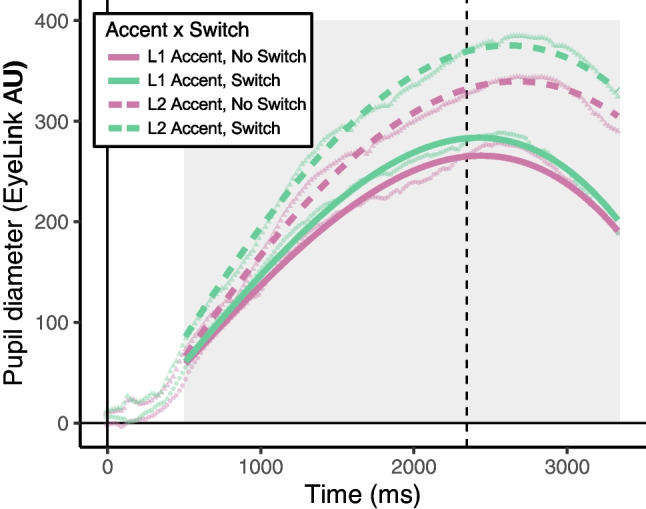
The Experiment 1 interaction between accent and switch is shown with model fits (lines) and raw data means (points). Y-axis shows pupil diameter in EyeLink AU (Arbitrary Units), where zero is the baseline calculated to align data across trials. X-axis shows time in milliseconds, beginning at trial start (zero). The dashed vertical line indicates the average offset time for all stimuli. The gray box indicates the window of the data used in analyses. Note that the accent conditions listed are from the current trial; thus, “L1 Accent, Switch” indicates a switch from L2 to L1 accent, and “L2 Accent, Switch” indicates a switch from L1 to L2 accent

The fixed effects of accent (reference level: L1 accent) and switch (reference level: no switch) were both dummy-coded. Accent had a significant effect on the intercept (β = 41.97, *p* < .001) and linear time terms (β = 329.00, *p* < .001), indicating a condition-wise difference in the overall peak of the pupillometry functions as well as in the rate of pupil size increase, respectively. Switch had a significant effect on the intercept (β = 9.70, *p* < .001) and quadratic terms (β = -0.48, *p* < .001). The direction of the accent estimate indicated that the L2 accent condition elicited relatively larger pupil response than the L1 accent condition, and the direction of the switch estimate indicated a larger pupil response for switches as compared to repeats. Notably, the interaction between accent and switch also significantly improved model fit (*p* < .001), indicating that the effect of switching speakers was larger for the L2 accent condition (Fig. 1; dashed lines vs. solid lines). Switching from the L1 to the L2 speaker (line labeled “L2 Accent, Switch”) was costlier than repeating the same L2 speaker (“L2 Accent, No Switch”), and costlier than switching from an L2 to an L1 speaker (“L1 Accent, Switch”). Post hoc tests were conducted to examine the effect of switch separately in datasets containing only L1 versus L2 trials. Results confirmed that the effect of switch was significant in both the L2 dataset (β = 24.51, *p* < .001) and the L1 dataset (β = 12.85, *p* < .001).

### Discussion

The results of Experiment 1 suggest that switching between speakers of different accents is more cognitively demanding than listening to the same speaker consecutively. Additionally, there is an asymmetry in the switch costs, where switching from an L1 to an L2 accent is particularly costly. While these results provide initial evidence of dynamic trial-to-trial processing adjustments to speakers and accents and a familiarity benefit, they are limited by the design of McLaughlin and Van Engen (2020).

## Experiment 2

In Experiment 2, we aimed to replicate and extend Experiment 1 with three key changes. First, we eliminated the subjective effort ratings, which were not relevant to the current aims.^2^ Second, we increased the number of speakers in Experiment 2 (two L1, two L2), allowing us to compare switch costs within and across accents. Third, rather than randomizing speakers, we designed our blocks with an equal number of trials with no switches, within-accent switches, and across-accent switches.

Our predictions are consistent with Experiment 1: Speaker switches should be more difficult than speaker repetitions, across-accent switches should be more difficult than within-accent switches, and an asymmetry should emerge such that switching from an L1 to an L2 accent is more demanding than switching from an L2 to an L1 accent.

### Method

Pre-registration, materials, experiment, data, and analysis code are all available from https://osf.io/ajmqz. The recruitment plan and protocol for this experiment was approved by the Washington University in St. Louis Institutional Review Board.

### Participants

Sixty-three young adult participants (46 female, 17 male; *M*_*age*_ = 19.68 years, *SD* = 1.10 years) were recruited from the Washington University Psychology Participants Pool. Recruitment for the study began before the COVID-19 pandemic, with approximately half of the subjects (*n* = 28) participating in 2020 (before campus closure), and the other half (*n* = 35) participating in 2021. Details regarding how procedures were changed to meet COVID-19 safety standards are discussed below. We report an exploratory analysis comparing data collected before and after campus shut down in the Online Supplemental Materials (OSM).

Recruiting subjects in the spring of 2021 proved more difficult, so we made two alterations to our pre-registered plans for recruitment and exclusions. First, we began offering cash payment as an additional option in place of course credit. Most subjects (*n* = 51) were compensated with course credit, and a small subset opted for a US$10 cash payment (*n* = 12). Additionally, in order to retain more subjects, we decided to only remove subjects with more than 20% of trials lost due to blinking (not 20% of trials lost due to blinking and incorrect responses combined).

After replacing subjects who were excluded due to experiment or equipment malfunction (11 subjects) and blinking-related data loss (two subjects), we met our target sample size of 50 participants (38 female, 12 male; *M*_*age*_ = 19.62 years, *SD* = 1.09 years). The sample size for Experiment 2 was selected based on sufficient power to detect effects in Experiment 1. All subjects were L1 speakers of English with normal hearing, normal (or corrected-to-normal) vision, and minimal exposure to Mandarin Chinese.

### Materials

Stimuli for Experiment 2 included recordings of two L1 American-English speakers and two Mandarin Chinese-accented speakers of English reading sentences with four keywords each (from the same sentence set as McLaughlin & Van Engen, 2020; Van Engen et al., 2012).^3^ All of the speakers were female. Neither of the speakers from McLaughlin and Van Engen (2020) were included. The two L2 speakers were selected from a set of three speakers (all L1 speakers of Mandarin) after an online transcription pilot. The design of the pilot was multi-talker, with files from the three talkers intermixed randomly. Approximately ten subjects provided a response for each item. Fifty-one sentences were presented to each subject, with no targets repeating. Multiple iterations of the pilot were conducted in order to collect data for 153 sentences. L2 Speaker 1 was found to be 93% intelligible in quiet (*SD* = 9%) and L2 Speaker 2 was found to be 94% intelligible in quiet (*SD* = 8%).

When examining the time-course of listening effort with pupillometry, it is important to match speaking rate across conditions (McLaughlin & Van Engen, 2020). Thus, the L2 speakers were instructed to read naturally while the L1 speakers were instructed to read slightly slower than natural. The set of recordings included 153 target sentences per speaker with pilot data, and when selecting target sentences for the present experiment (which required 104 targets), we matched the average lengths of target files across speakers (2,860 ms). We also aimed to select the sentences with the highest intelligibility across the two L2 speakers.^4^

### Procedure

Participants entered the lab and confirmed that they were L1 English speakers, did not have extensive exposure to Mandarin-accented speech (e.g., living with a Mandarin speaker, studying Mandarin), had normal hearing, and had normal or corrected-to-normal vision. For pre-COVID participants, the experimenter brought them to a testing room and began the instructions. For the COVID protocol, participants were instructed to enter the testing room and instructions were delivered via video call. The trial procedure was adapted from McLaughlin and Van Engen (2020).

Participants wore circumaural headphones and rested their chins on a head-mount that was 90 cm away from a 53.5 cm × 30 cm computer screen. All equipment was positioned following EyeLink specifications. A nine-point calibration and validation procedure was conducted for all subjects before they began the task.

During the task, participants were instructed to fixate on a cross located in the center of the screen. When the cross was red, participants were instructed to reduce blinking as much as was comfortable and to attend to the auditory stimulus. When the cross was blue, participants were instructed to blink freely. Each trial began with a baseline period of 3,000 ms of silence and a red cross. Next, with the red cross still present, the stimulus played followed by a delay period of 3,000 ms. At this point, the color of the cross turned to blue, indicating that subjects could blink freely. Participants were instructed to repeat what they heard aloud. For pre-COVID participants, responses were recorded with an audio recorder. For COVID participants, responses were recorded as part of the ongoing video call. Finally, participants pressed the spacebar to move to the next trial, and a 3,000-ms silent delay period with a blue fixation cross was presented. This delay allowed the pupil response to recover between trials.

Participants began with four practice trials, one per speaker. These practice trials followed the same trial procedure as the experimental task. Next, subjects completed the four 25-trial experimental blocks. Each block began with a start trial, which was not included in our analyses because it was neither a repeat nor a switch trial. Each of the four speakers was the start trial in one of the four blocks. Of the remaining 24 trials per block, each of the four speakers was presented six times. The order of trials was unpredictable from the participant’s perspective, but was pseudorandom so that the lists contained the same number (eight each) of the key transition types: no switch, within-accent switch, and across-accent switch. For no switch transitions, the speaker from trial N-1 spoke on trial N (i.e., the current trial). For within-accent switches, the speaker on trial N-1 had the same accent as the speaker on trial N, but was a different speaker. Lastly, for across-accent switches, the speaker on trial N-1 was a different speaker with a different accent. Additionally, for each of the three main conditions, we considered the accent of the speaker on trial N. We compared, for example, an across-accent switch from L1 to L2 with an across-accent switch from L2 to L1. Between each block, there was a self-timed break. Subjects were instructed not to leave their chair or remove their head from the chinrest during these breaks.

After completing the four experiment blocks, participants completed language and demographic questionnaires. Finally, participants were debriefed on the task. The entire procedure took approximately 45 min.

### Data preparation

Repetitions of the target sentences were scored to determine whether subjects correctly understood the speaker. Each sentence had four keywords (e.g., the *gray mouse ate* the *cheese*). Any trial in which any keyword was misidentified or missing was excluded from analyses. Differences in plurality and verb tense (specifically differences in use of -ed morpheme) were allowed. In the L2 accent condition, 7.7% of trials were removed for being < 100% intelligible (or in some cases, due to poor recording quality that prevented response scoring). In the L1 accent condition, only 1.2% of trials were lost.

For the pupil data, pre-processing was completed using the R package gazeR (Geller, et al., 2020). Subjects with more than 20% data loss due to blinking were excluded (two subjects). Periods of missing data due to blinks were next identified and extended 100 ms prior and 200 ms following. This process removes extraneous values that occur when the eyelid is partially obscuring the pupil. For these extended blink windows, linear interpolation was used to fill in the missing data. A five-point moving average then smoothed the data. The median pupil diameter during the 500 ms immediately preceding stimulus onset was used as the baselining value for each trial. Subtractive baselining was used (Reilly et al., 2019). As a final step, the data were time-binned, reducing the sampling frequency from 500 Hz to 50 Hz.

### Analysis window selection

Time window selection for growth curve analysis can increase researcher degrees of freedom during the analysis process (Peelle & Van Engen, 2021). To avoid biasing our analyses, we selected our analysis window without viewing its influence on the effects of interest. The only data viewed prior to window selection was a single plotted curve summarizing the mean of all trial and subject data (i.e., to confirm a polynomial analysis was appropriate). Due to the delay of the pupil response, which is typically 200–300 ms, we opted to begin our analysis window at 300 ms after target onset. The end of the analysis window was based on the average offset time of the stimuli (2,860 ms).

### Results

The random-effect structure matched that of Experiment 1.^5^ Table 2 summarizes all log-likelihood model comparisons from the growth curve analysis of the full dataset. The linear, quadratic, and cubic polynomials all significantly improved fit (all *p*s < .001). Given the complex shape of the pupil response, we also tested whether a quartic polynomial would improve fit. It did not (χ^2^(1) = 0.02, *p* = .88), and thus was not retained in subsequent models.

**Table 2:** Log-likelihood model comparisons for growth curve analysis of Experiment 2

Effect	*χ*^2^	Df	*p*
Linear polynomial	3074.30	1	< .001 ***
Quadratic polynomial	1147.70	1	< .001 ***
Cubic polynomial	112.64	1	< .001 ***
Quartic polynomial*	0.02	1	.88
Accent (Levels: L1 Accent, L2 Accent)	2877.50	1	< .001 ***
Switch (Levels: No Switch, Within-accent Switch, Across-accent Switch)	272.91	1	< .001 ***
Accent × Linear polynomial	2467.20	1	< .001 ***
Accent × Quadratic polynomial	37.99	1	< .001 ***
Accent × Cubic polynomial	26.41	1	< .001 ***
Switch × Linear polynomial	28.96	2	< .001 ***
Switch × Quadratic polynomial	16.40	2	< .001 ***
Switch × Cubic polynomial	3.82	2	.15
Accent × Switch	109.34	2	< .001 ***

*Effect not retained in subsequent models

**Fig. 2:**
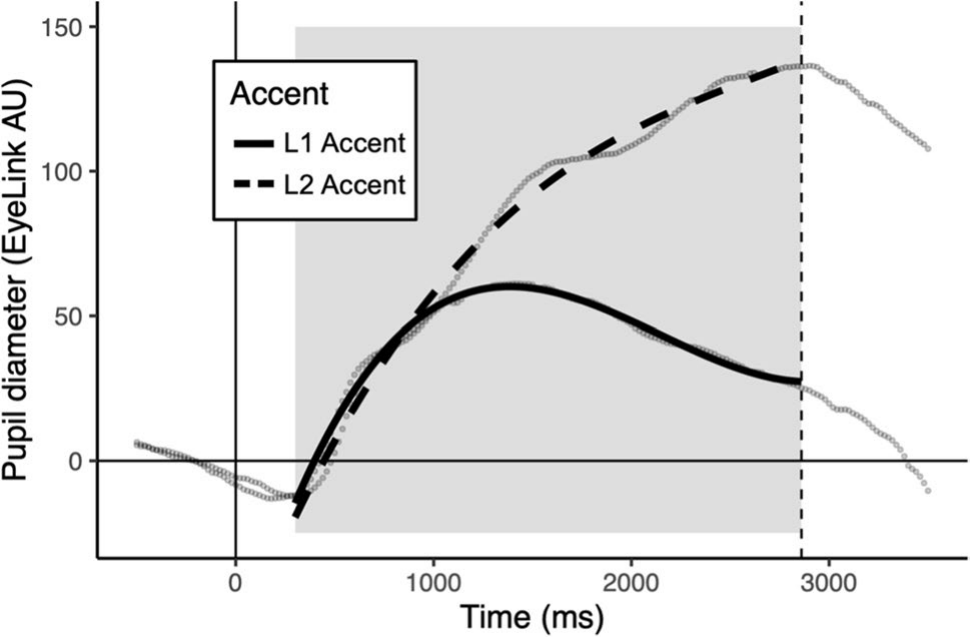
The effect of accent on the size of the pupil in Experiment 2 is shown with model fits and raw data points. The y-axis shows pupil diameter in EyeLink AU (Arbitrary Units), where zero is the baseline calculated to align data across trials. The x-axis shows time in milliseconds, beginning at trial start (zero). The dashed vertical line indicates the average offset time for all stimuli. The gray box indicates the window of the data used in analyses

**Fig. 3:**
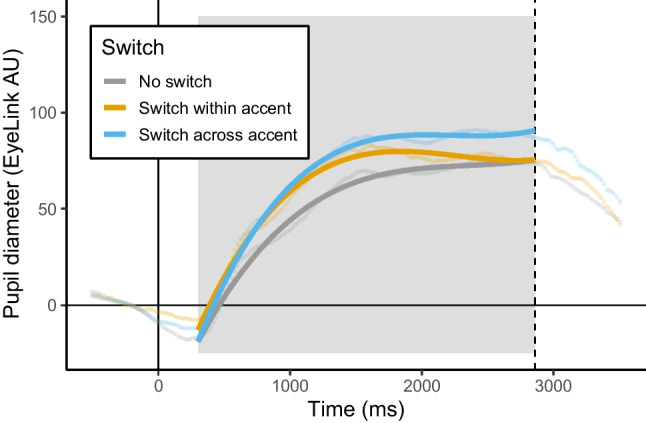
The effect of switch on the size of the pupil in Experiment 2 is shown with model fits and raw data points. The y-axis shows pupil diameter in EyeLink AU (Arbitrary Units), where zero is the baseline calculated to align data across trials. The x-axis shows time in milliseconds, beginning at trial start (zero). The dashed vertical line indicates the average offset time for all stimuli. The gray box indicates the window of the data used in analyses

First, we examined the dummy-coded main effects of accent (reference level: L1) and switch (reference level: no switch). Accent had a significant effect on the intercept (β = 42.17, *p* < .001), linear term (β = 441.89, *p* < .001), quadratic term (β = 54.78, *p* < .001), and cubic term (β = -45.67, *p* < .001). These effects indicate a condition-wise difference in the overall peak, rate, and shape of the pupil response. As in Experiment 1, pupil response was larger for L2-accented compared to L1-accented speech (Fig. 2). The contrast of the no switch and within-accent switch conditions revealed a significant effect on the intercept (β = 9.44, *p* < .001), linear term (β = -48.07, *p* < .001), and quadratic terms (β = -34.57, *p* = .002), whereas the contrast of the no switch and across-accent switch conditions revealed a significant effect on the intercept (β = 8.20, *p* < .001) and quadratic terms (β = -41.47, *p* < .001) only (although the effect on the cubic polynomial was also marginal: β = 20.65, *p* = .06). Altogether, these outcomes indicate a larger pupil response for switching speakers within and across accent as compared to listening to the same speaker consecutively. As visualized in Fig. 3, the shape of the model fit indicates a larger early rise of the pupil response for the switching conditions; however, the shape of the pupil response within- and across-accent switches diverges near sentence-offset.

**Fig. 4:**
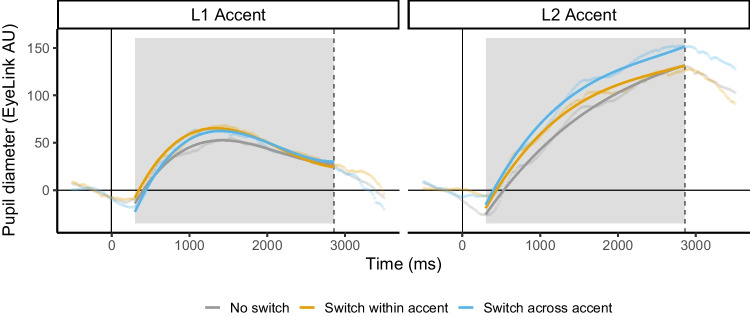
The interaction between the effects of accent and switch in Experiment 2 is captured in two panels, with the labels indicating the accent of the current trial. Note that the across-accent switch line (blue) in the left panel indicates a switch from L2 to L1 accent, and this line in the right panel indicates a switch from L1 to L2 accent. For both panels, the y-axis shows pupil diameter in EyeLink AU (Arbitrary Units), where zero is the baseline calculated to align data across trials. The x-axis shows time in milliseconds, beginning at trial start (zero). The dashed vertical line indicates the average offset time for all stimuli. The gray box indicates the window of the data used in analyses. For an enlarged version these same growth curve model fits with standard errors, please see Online Supplemental Material Figs. 2 and 3

Adding the interaction between accent and switch significantly improved model fit (χ^2^(2) = 109.34, *p* < .001), but model estimates indicated that there was no difference in the cognitive demands for the within-accent switch condition (as compared to the no switch condition) for L1 and L2 accents (β = -1.99, *p* = .31). Rather, this interaction appears to be driven by across-accent switching, for which pupil response is greater when switching from L1 to L2 accent (β = 16.32, *p* < .001; blue line of right panel of Fig. 4).

To examine the cognitive demands for within- versus across-accent switching, we reordered the levels of switch to make the within-accent switch condition the reference level of the factor.^6^ When examining a model without interactions, model estimates indicated that pupil response was greater for switching speakers across-accent than within-accent (β = 7.69, *p* < .001; Fig. 3). Additionally, when examining a model with all of the fixed effects including interactions, model estimates from the accent by switch interaction indicated that the difference in pupil response between the across-accent and within-accent conditions was larger for the L2 than the L1 accent condition (β = 18.31, *p* < .001; Fig. 4).

We also pre-registered follow-up analyses to separately examine switch costs for the L1 and L2 accent conditions. For the L1 accent condition, log-likelihood model comparisons indicated that switch improved model fit (χ^2^(2) = 58.09, *p* < .001). Model estimates indicated that pupil response for switching both within- (β = 8.70, *p* < .001) and across-accent (β = 8.65, *p* < .001) was greater than pupil response for not switching, but there was no difference in pupil response for within- and across-accent switching (β = -0.04, *p* = .97; Fig. 4).

Finally, we completed this process again for the L2 accent data. Switch also improved model fit (χ^2^(2) = 388.17, *p* < .001), and model estimates indicated that all levels differed from each other (*p*s < .001).

### Discussion

The results of Experiment 2 replicate and extend the findings of Experiment 1. As predicted, trials with speaker switches were more difficult than those with repetitions, across-accent switches were more difficult than within-accent switches, and an asymmetry emerged – switching from an L1 to an L2 accent was more demanding than switching from an L2 to an L1 accent.

## General discussion

Using pupillometry as an online measure of cognitive effort, we present novel evidence that switching between speakers universally increases demand. Additionally, switching speakers across accents increases demand more than switching speakers within an accent. This effect differed based on the accent of the current trial’s speaker, such that switching from an L1 to an L2 speaker was more costly than switching between two L2 speakers whereas switching from an L2 to an L1 speaker was no more costly than switching between two L1 speakers. These results reveal that listeners make dynamic and cognitively effortful adjustments trial-to-trial in response to different speakers.

The within-accent switch costs observed in the present study can be accounted for by both an active control model and an auditory streaming framework. However, the increased demands associated with across-accent switches compared to within-accent switches cannot be accounted for by an auditory streaming framework alone.^7^ A key aspect of the auditory streaming framework is that disruption of auditory attention should be consistent across all speaker switches, regardless of accent. To accommodate our findings within the auditory streaming framework, we would need to assume that two mechanisms (e.g., selective auditory attention and talker normalization) operate in parallel for multi-talker listening conditions (consistent with Choi et al., 2022). The within-accent switch costs in the present study may reflect the disruption of auditory attention, while the across-accent switch costs may reflect the active engagement of a talker normalization mechanism.

The asymmetry in across-accent switch costs indicates that a familiarity benefit is afforded to L1-accented speech. Talker normalization, exemplar, and non-analytic episodic theories all predict that familiar input should pose lesser processing costs for the listener. In line with these frameworks, our data suggest that switching from an L2- to an L1-accented speaker was no more difficult than switching from one L1-accented speaker to the other. Thus, a stable speaker-switching cost occurs, but no additional cost occurs for making the relatively larger shift in “phonetic distance” between the two speakers of different accents. In contrast, switching from an L1- to an L2-accented speaker is more cognitively demanding than switching between two L2-accented speakers. As no familiarity benefit is afforded, the processing costs associated with switching to an unfamiliar L2 accent are much greater.

A related but distinct way to conceptualize these trial-by-trial adjustments is based on differences in the upregulation of cognitive control^8^ (cf. Botvinick et al., 2001). Here, one can ask whether the sequential adjustments are proactive (i.e., the accent on trial N-1 heightens control that is actively maintained and influences trial N) or reactive (i.e., the heightening of control might residually carry over to the next trial, with such carry over anticipated to decay at longer intervals; e.g., Scherbaum et al., 2012). CSEs have been interpreted as reactive because they are diminished or eliminated for “long” ISIs between 2,250 and 5,000 ms (Egner et al., 2010; but see Duthoo et al., 2014b). The sequence effects in the present study were observed using much longer ISIs (10–20 s), favoring a proactive account. However, one should also consider a distinct reactive mechanism that persists across long delays and intervening trials. Participants might associate a stimulus feature (e.g., accent) on trial N-1 with the degree of control used on that trial (e.g., L2 = high control), and the associated degree of control might be reactivated when encountering the accent on trial N (i.e., a CSE learning account; Freund & Nozari, 2018). A key avenue for future research will be systematically examining how the sequential adjustments that occur for multi-talker and multi-accent speech processing are affected by runs of the same speaker on previous trials and extend over longer ISIs and across subsequent trials (e.g., trial N+1, trial N+2, etc.).

One of the limitations of the present work is the limited number of talkers and accents included in the experimental design (two each). Although we provide some positive evidence of a familiarity benefit, we did not parametrically vary accent familiarity (e.g., familiar vs. unfamiliar L2 accent). Further work with additional speakers and accent varieties of varying familiarities will be needed to determine the generalizability of these findings.

## Conclusion

The challenge listeners face when mapping acoustic input onto linguistic representations can be complicated by both speaker and accent variability. In the present study, we used pupillometry to track cognitive processing load for trial-to-trial switches between speakers of the same or different accents. Our results indicated a universal cost for switching between speakers, and an asymmetry in the costs for switching between different accents. Switching from an L1 speaker to an L2 speaker was more cognitively demanding than switching between two L2 speakers, while the reverse (switching from an L2 to an L1 speaker) was no different than switching between two L1 speakers. These sequence effects observed for speech processing align with work examining multi-talker processing costs, and provide novel evidence that a familiarity benefit may be afforded to L1-accented speech in a multi-accent listening setting. Altogether, these findings align with an active control model, but may also support the conclusion that two mechanisms (an auditory streaming and talker normalization mechanism) work in parallel to support speech processing in multi-talker and multi-accent settings.

### Supplementary Information

Below is the link to the electronic supplementary material.

Supplementary file1 (DOCX 1001 KB)

## Data Availability

The datasets generated and/or analyzed during the current study are available via the Open Science Framework repository at: https://osf.io/nzgyb/files/.

## Footnotes

1 The key difference between exemplar theory and non-analytic episodes theory is that the former proposes that features are abstracted from episodes in memory, while the latter proposes that episodes remain whole and unabstracted in memory. For the purposes of the present paper, we discuss these theories jointly.

2 The analysis in Experiment 1 assumed that any interference of this measure (which was acquired every three trials) with the sequence effects data was equally spread across conditions. By removing this feature entirely from Experiment 2, we were able to confirm this assumption and remove a potential source of noise from the task.

3 While we chose Mandarin as the non-native accent in the current study, it is important to note that non-native accents are not homogenous. The effects observed in the present study using American English and Mandarin may not be representative of all accents. It remains an open question whether similar patterns will be observed for across-accent switching between different native and non-native accents.

4 It should be noted that we could not examine sequence effects in task performance (e.g., response time (RT) or accuracy) because the present paradigm requires high levels of intelligibility for all speakers. These kinds of sequential adjustments remain an intriguing avenue for future research.

5 As the fixed effects in the model grew more complex, it became more difficult for models to converge and we had to simplify the random effect structure by removing the polynomial random slopes nested with items.

6 Note that changing the reference level does not alter model fit. The only change to the model is the estimates, allowing us to directly compare additional conditions of interest.

7 There is some evidence that may indicate that disruption of auditory streaming is less binary in nature (see Stilp & Theodore, 2020).

8 In the CSE literature, researchers have distinguished accounts based on bottom-up associative mechanisms (e.g., benefit is observed for consecutive incongruent trials because stimulus and/or response features repeat; e.g., Mayr et al., 2001) from control mechanisms (i.e., benefit is observed because the conflict-triggered heightening of attention toward the goal-relevant dimension on N-1 benefits trial N; e.g., Botvinick et al., 2001). Given the current study’s use of unique sentences and responses on each trial, an account based on bottom-up associative mechanisms appears unlikely. Rather, a control-based account is more plausible.

## Code availability

The code used to analyze data from the current study are available via the Open Science Framework repository at: https://osf.io/nzgyb/files/.
