# Supplementary material for: Sequence effects and speech processing: cognitive load for speaker-switching within and across accents
Source: Psychon Bull Rev. 2023 Jul 13;31(1):176–86. doi: 10.3758/s13423-023-02322-1 (PMC10867039; doi:10.3758/s13423-023-02322-1)
Supplement: Supplementary file 1 — Supplementary file1 (DOCX 1001 KB) [file 13423_2023_2322_MOESM1_ESM.docx]

**Supplemental Materials**

**1. Pandemic effects**

As part of our visual inspection of the data, we compared performance of subjects who participated in the study before the onset of the pandemic (“Pre-COVID”; *n* = 22) versus subjects who participated after the onset of the pandemic (“Post-COVID”; *n* = 28). Supplemental Figure 1 shows the overall difference between these two groups and individual subject trends. A basic linear mixed-effects model with random intercepts for subjects indicated that subjects who participated after the onset of the pandemic had larger overall pupil response during the task (*p* = .04).

**Supplemental Figure 1**

*Time of Participation Relative to the Onset of the Pandemic*


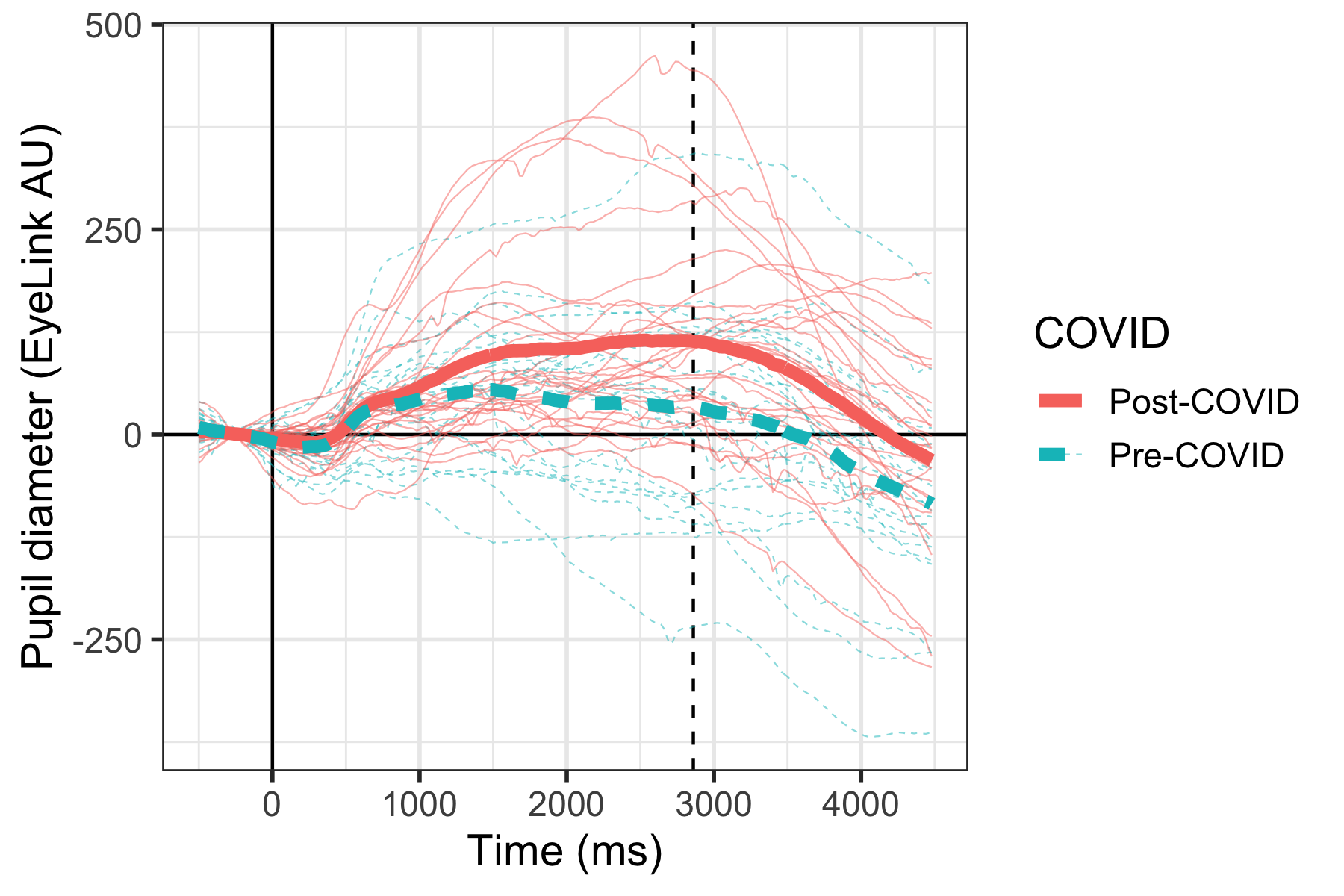


*Note.* Overall pupil response during the task is plotted based on time of participation relative to the COVID-19 pandemic. The y-axis shows the size of the pupil over time within a trial (x-axis). Thick lines show group means, and thin lines show individual subjects’ mean curves. The solid vertical line indicates the beginning of stimulus presentation and the dashed vertical line indicates the average offset of stimulus presentation.

The difference between the subjects who participated before and after the onset of the COVID-19 pandemic is surprising when considering that our equipment and experiment remained identical. One possible explanation for the data is the change to our procedures for COVID-related safety. When data collection resumed (i.e., after the suspension of in-person data collection due to COVID-19 had been lifted), we changed our procedures in the following ways: Subjects were required to wear masks at all times, including when completing the task and resting their chin on the pupillometry head mount; subjects did not interact with the researcher directly, but were communicated with via a Zoom call on a computer added to the pupillometry testing suite; and subjects were told that the Zoom call would run throughout the entire experiment, and that the researcher could hear them if they needed assistance at any point. It is reasonable to suggest that these factors could have affected engagement with the task.

Another explanation for the effect of the pandemic on the data would be differences in subjects’ stress and arousal levels. Given that the pupil is affected by stress and arousal, it is plausible that subjects entered the experiment session in a heightened state of stress unrelated to the task itself (for example, worrying about coming into contact with someone sick). Indeed, for many of the “Post-COVID” subjects in the study it was likely one of their first outings since the beginning of the COVID-19 pandemic. In either case, this change could have affected pupil response during the task, resulting in larger overall pupil responses for subjects participating after the onset of the pandemic.

Although the effects of the pandemic on the current data are certainly notable, we caution against drawing conclusions from these data because of the smaller sample size in this between-subject comparison. We report these data here for the purposes of transparency as well as to alert other researchers who have used pupillometry throughout the onset and continuation of the pandemic.

**2. Additional figures**

**Supplemental Figure 2**

*Growth curve model fits for switching effect in L1 accent condition*


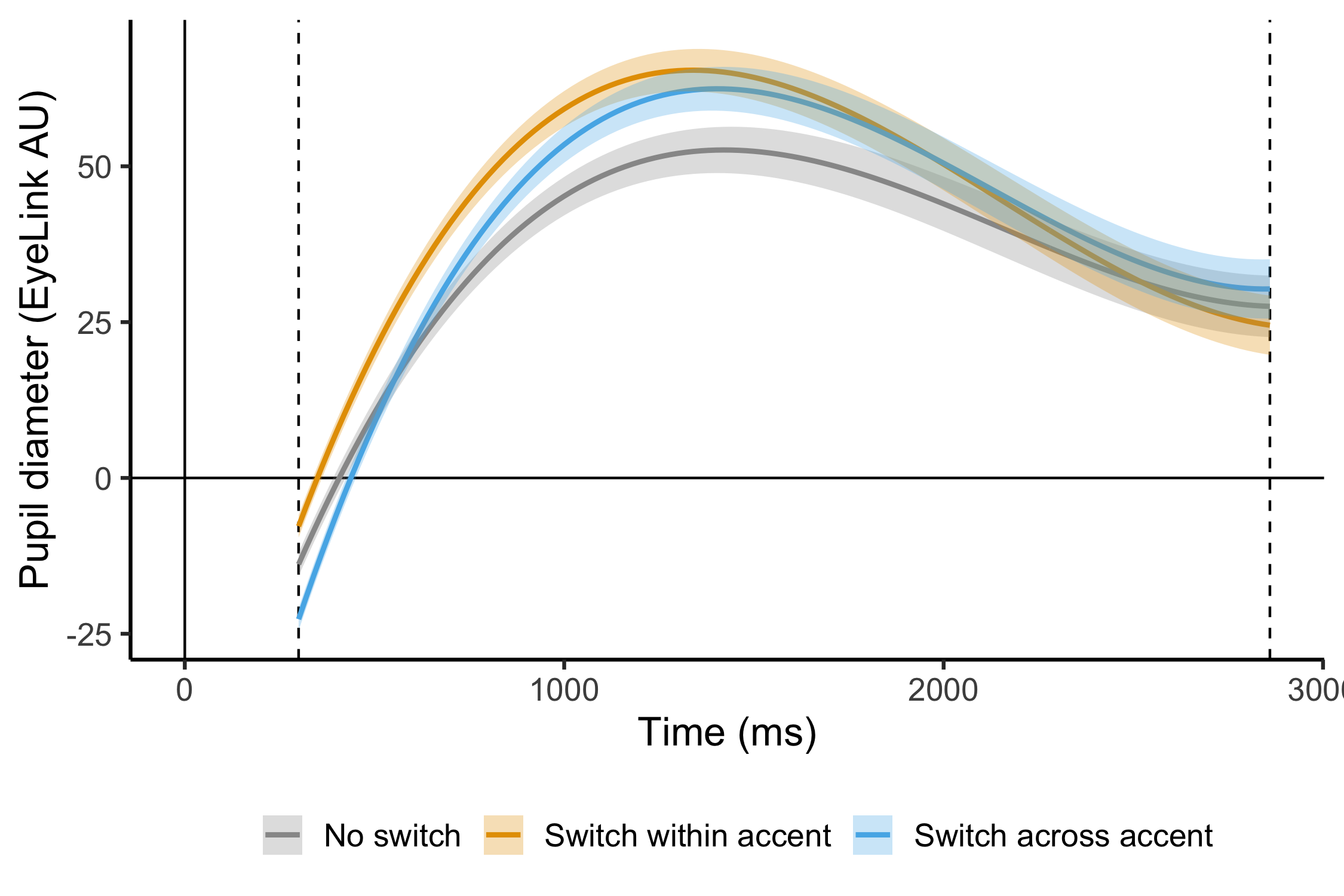


*Note.* Growth curve analysis model fits and standard errors are shown for each level of the effect of switch in the L1 accent condition. On the x-axis, zero indicates the beginning of a trial. The beginning of the window of interest used for analyses (i.e., the onset of sentences) is marked with the leftmost dashed vertical line, and the end of this window is marked with the rightmost vertical line. On the y-axis, zero indicates baseline.

**Supplemental Figure 3**

*Growth curve model fits for switching effect in L2 accent condition*


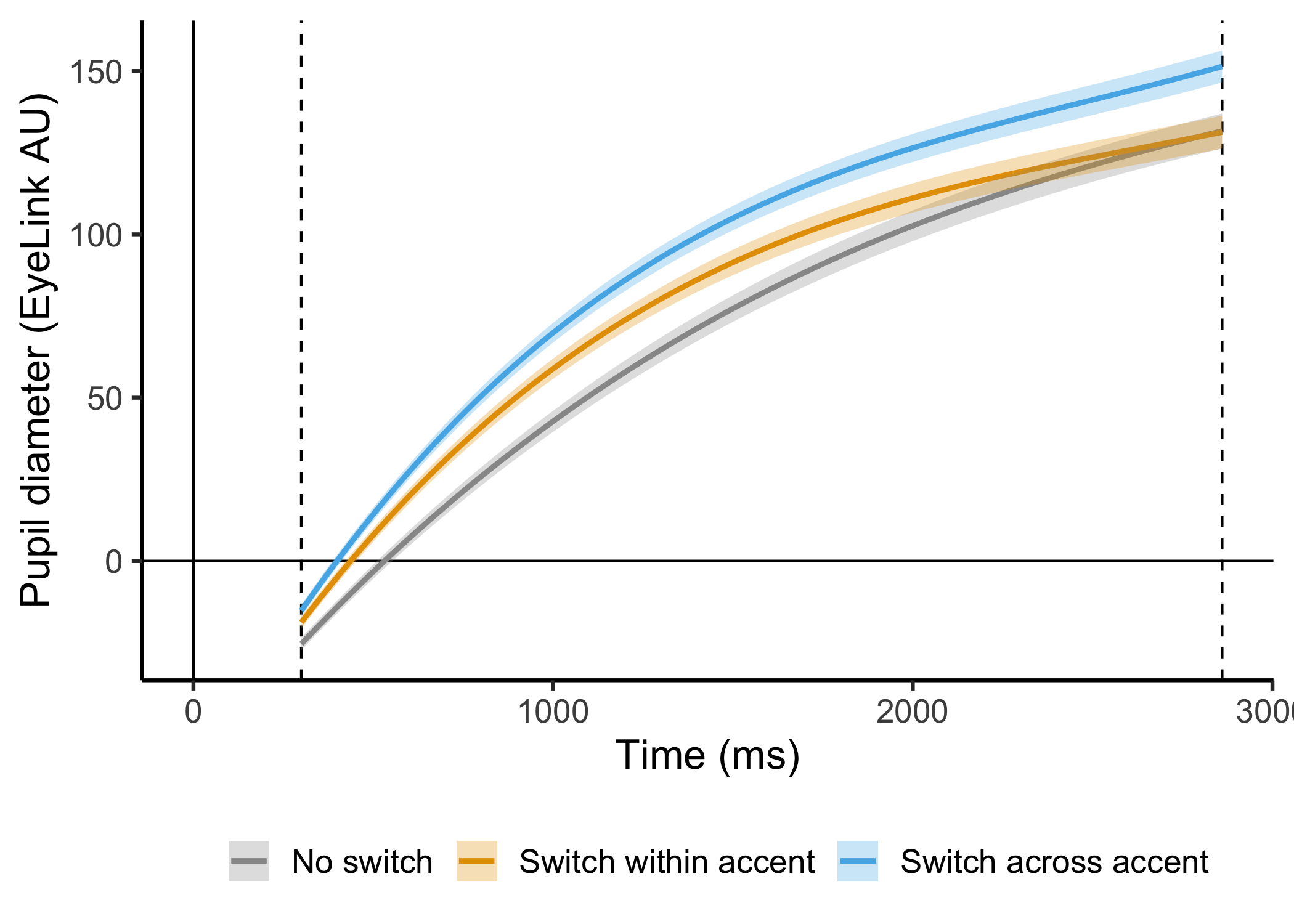


*Note.* Growth curve analysis model fits and standard errors are shown for each level of the effect of switch in the L2 accent condition. On the x-axis, zero indicates the beginning of a trial. The beginning of the window of interest used for analyses (i.e., the onset of sentences) is marked with the leftmost dashed vertical line, and the end of this window is marked with the rightmost vertical line. On the y-axis, zero indicates baseline.
